# Bio-assisted synthesized Pd nanoparticles supported on ionic liquid decorated magnetic halloysite: an efficient catalyst for degradation of dyes

**DOI:** 10.1038/s41598-020-63558-8

**Published:** 2020-04-16

**Authors:** Samahe Sadjadi, Pourya Mohammadi, Majid Heravi

**Affiliations:** 1 Faculty of Petrochemicals, Iran polymer and Petrochemicals Institute, 15 km Tehran-Karaj Highway, Pajuhesh Science and Technology Park, Pajuhesh Boulevard, postal cod; 14977-13115, PO Box 14975-112, Tehran, Iran; 2 Department of Chemistry, School of Science, Alzahra University, PO Box 1993891176, Vanak, Tehran Iran

**Keywords:** Chemistry, Catalysis, Environmental chemistry, Green chemistry

## Abstract

Using natural materials, i.e. halloysite nanoclay that is a biocompatible naturally occurring clay and Heracleum persicum extract that can serve as a green reducing agent, a novel magnetic catalyst, Fe_3_O_4_/Hal-Mel-TEA(IL)-Pd, has been designed and fabricated. To prepare the catalyst, halloysite was first magnetized (magnetic particles with mean diameter of 13.06 ± 3.1 nm) and then surface functionalized with melamine, 1,4 dibromobutane and triethanolamine to provide ionic liquid on the halloysite surface (5 wt%). The latter was then used as a support to immobilize Pd nanoparticles that were reduced by Heracleum persicum extract. The characterization of the catalyst established that the loading of Pd in Fe_3_O_4_/Hal-Mel-TEA(IL)-Pd was very low (0.93 wt%) and its specific surface area was 63 m^2^g^−1^. Moreover, the catalyst showed magnetic property (Ms = 19.75 emu g^−1^) and could be magnetically separated from the reaction. The catalytic performance of the magnetic catalyst for reductive degradation of methyl orange and rhodamine B in the presence of NaBH_4_ in aqueous media was investigated. The activation energy, enthalpy, and entropy for the reduction of methyl orange were estimated as 42.02 kJ mol^−1^, 39.40 kJ mol^−1^, and −139.06 J mol^−1^ K^−1^, respectively. These values for rhodamine B were calculated as 39.97 kJ mol^−1^, 34.33 kJ mol^−1^, and −155.18 Jmol^−1^K^−1^, respectively. Notably, Fe_3_O_4_/Hal-Mel-TEA(IL)-Pd could be reused for eight reaction runs with negligible loss of the catalytic activity (~3%) and Pd leaching (0.01 wt% of the initial loading).

## Introduction

Application of low-cost, biocompatible and available natural compounds for the catalytic purposes has attracted tremendous attention. In this regard, use of halloysite (Hal) that is a dioctahedral 1:1 clay of the kaolin group has received growing interest^1–3^. Apart from high thermal, chemical and mechanical stability, the  cylindrical morphology and opposite chemical and electrical properties of inner and outer surfaces of Hal, make it distinguished from other clays and broaden its utility for many applications such as cleaning^4^, adsorbents^5,6^, energy storage, catalysis, flame retardancy^1^, sustainable release of drugs etc. Regarding catalysis, Hal not only can be utilized as a catalyst, but also can be applied as a support for the development of reusable catalysts. To date, various chemical, photochemical and electrochemical transformations such as coupling reactions^7,8^, hydrogenation^9,10^, oxidation have been promoted by using Hal-based catalysts^11–15^. Organic dyes such as methylene blue, rhodamine B (RhB) and methyl orange (MO) are toxic chemicals with potential health threat^16^. The release of these compounds in the industrial waste water has raised many concerns and motivated many studies for the efficient removal of these hazardous pollutants or their conversion into less toxic compounds^17,18^. In this context, several approaches, such as use of adsorbents, photodegradation, electrochemical degradations, membrane processes, oxidative processes and catalytic reduction of dyes have been developed^19–21^. Dye reduction is a catalytic decolorization process that is mostly promoted by metallic catalysts such as Pd, Pt and Au and a reducing agent such as NaBH_4_^22^.

**Figure 1:**
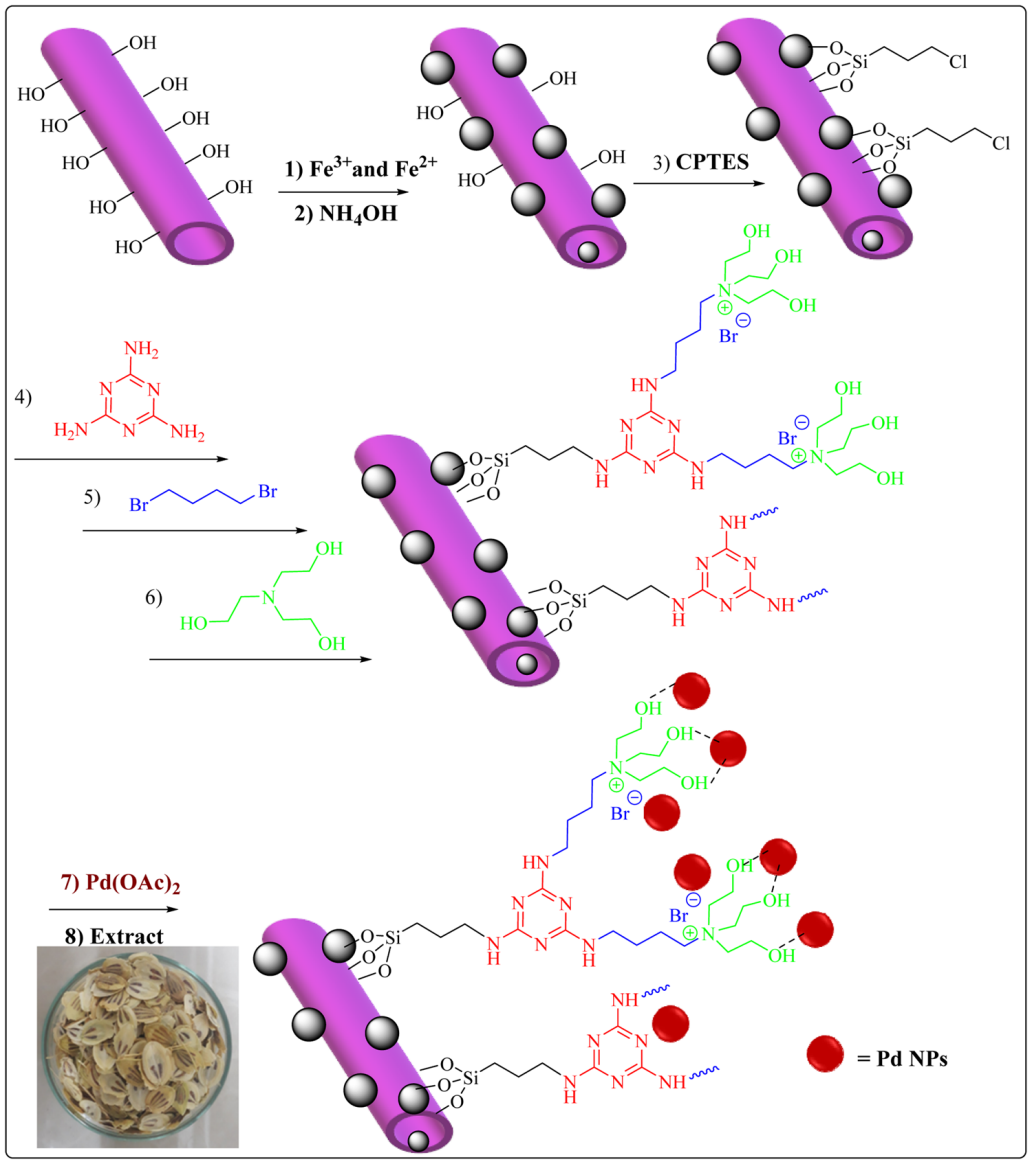
The schematic procedure for the synthesis of the catalyst.

In the continuation of our research on disclosing the catalytic utility of Hal^23,24^, recently we have reported facile recovery and high reusability of magnetic Hal^25,26^. On the other hand, our research on the catalytic activity of IL-Hal hybrid^27,28^ showed that the presence of ILs on the Hal can efficiently improve anchoring of nanoparticles and suppressing their leaching. In this work we design and synthesize a novel catalyst through multi-step procedure, Fig. 1, in which Hal was first magnetized and then reacted with melamine and 1, 4 di bromobutane. The resulting compound then tolerated reaction with triethanolamine to form IL. In the final step, Pd nanoparticles were supported on the IL-decorated magnetic Hal with the aid of Heracleum persicum extract as a biological reducing agent. To evaluate the catalytic activity of the resulting catalyst, the reduction of two dyes MO and RhB was studied in aqueous media. The reusability of the catalyst was also investigated. Moreover, the kinetic parameters, including activation energy, entropy and anthalpy of reduction of each dye have been calculated.

## Results and discussions

### Characterization

**Figure 2:**
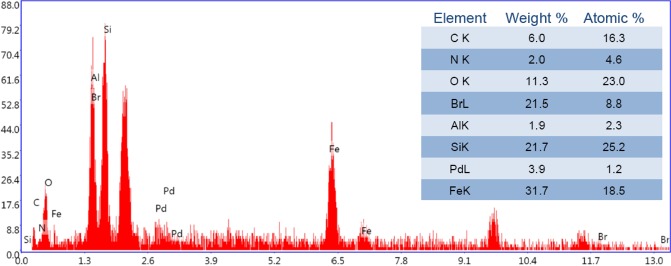
EDS analysis of the catalyst.

As depicted in Fig. 2, EDS analysis of the catalyst confirmed the presence of Al, Si and O atoms that can be attributed to the Hal structure. Notably, Si, O and C atoms can also represent the conjugation of CPTES. The presence of Fe atom indicated the successful magnetization of Hal. Observation of Pd atom also confirmed that Pd paricles were incorporated to the structure of the catalyst. The presence of C, N and Br atoms can be assigned to the organic moieties (melamine and IL). Figure S1 showed the elemental mapping analysis of Fe_3_O_4_/Hal-Mel-TEA(IL)-Pd. It was found that magnetic and Pd nanoparticles were well dispersed on the support.

**Figure 3:**
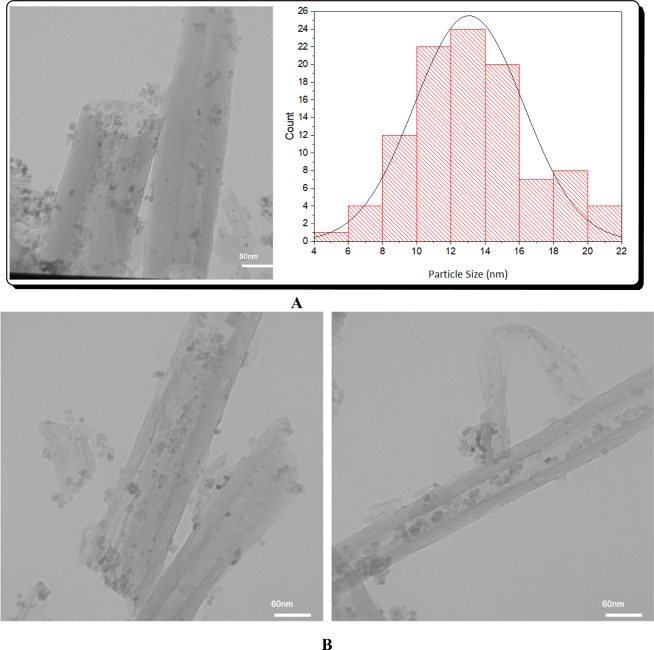
TEM image of Fe_3_O_4_/Hal (**A**) and TEM images of the catalyst (**B**).

In Fig. 3A the TEM image of Fe_3_O_4_/Hal is presented. As depicted, upon magnetization, Hal preserved its cylindrical morphology. Moreover, it can be seen that magnetic nanoparticles were located both on Hal exterior and interior surfaces. The measurement of the magnetic nanoparticle average size, Fig. 3A, showed that the synthetic procedure led to the formation of relatively small Fe_3_O_4_ nanoparticles (mean diameter of 13.06 nm with standard deviation of 3.1). The TEM analysis of the catalyst, Fig. 3B, also showed the cylinders of Hal, indicating that functionalization with organic moieties did not lead to the collapse of Hal structure. The dark black spots on the TEM image of the catalyst can be assigned to the magnetic and Pd nanoparticles.

**Figure 4:**
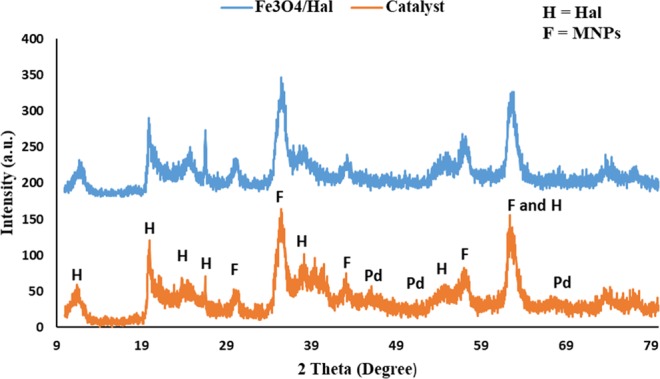
XRD patterns of the catalyst and magnetic Hal.

In the next step, the structure of the catalyst was studied by recording its XRD pattern and comparing it with that of Fe_3_O_4_/Hal. As depicted in Fig. 4, the XRD pattern of Fe_3_O_4_/Hal showed two series of characteristic bands, i.e. the characteristic bands of Hal and those of magnetic nanoparticles. According to the literature, the characteristic bands of Fe_3_O_4_ can be observed at 2θ = 30.52° (220), 35.5° (311), 43.18° (400), 57.22° (511), 62.73° (440) (denoted as F)^29^. The characteristic bands of Hal appeared at 2θ = 12.4°, 20.5°, 25.2°, 36.7°, 39.0°, 56.3° and 62.5° (the standard JCPDS card no. 29–1487, labeled as H)^30^. The XRD pattern of Fe_3_O_4_/Hal-Mel-TEA(IL)-Pd is very similar to that of Fe_3_O_4_/Hal and no displacement of the Hal and magnetic nanoparticles bands can be detected. This observation can indicate that decoration of magnetic Hal with melamine and IL as well as Pd incorporation did not affect the Hal structure and Hal maintained its tubular morphology. On the other hand, in the XRD pattern of Fe_3_O_4_/Hal-Mel-TEA(IL)-Pd some small additional bands at 2θ = 40 °, 51.6° and 68.4, JCPDS, Card No. 46–1043, labeled as Pd) can be observed that can be assigned to the Pd nanoparticles.

To study the magnetic feature of Fe_3_O_4_/Hal-Mel-TEA(IL)-Pd its magnetization curve was recorded and compared with that of bare magnetic nanoparticles, Fig. S2. The results showed that the magnetic saturation (Ms) value of Fe_3_O_4_/Hal-Mel-TEA(IL)-Pd (19.75 emu g^−1^) was far lower than that of bare Fe_3_O_4_ (47.1 emu g^−1^). It is worth noting that although the magnetic saturation of the catalyst was low, Fe_3_O_4_/Hal-Mel-TEA(IL)-Pd was magnetic enough to be readily separated from the reaction mixture by using an external magnet.

Next, the thermal stability of Fe_3_O_4_/Hal-Mel-TEA(IL)-Pd was studied by recording Fe_3_O_4_/Hal-Mel-TEA(IL)-Pd thermogram. Furthermore, to measure the content of IL and melamine, the thermograms of Hal and Fe_3_O_4_/Hal-Mel were also recorded and compared with that of Fe_3_O_4_/Hal-Mel-TEA(IL)-Pd, Fig. S3. Among three thermograms, Hal possessed the highest thermal stability and showed only two weight losses (loss of water below 200 °C and Hal dehydroxylation at about 470 °C). This is not beyond expectation, as bare Hal possesses no organic moieties. Followed by Hal, Fe_3_O_4_/Hal-Mel showed high thermal stability. In this sample, apart from the Hal weight losses, an additional weight loss at ~200 °C with weight loss of ~12 wt% is observed that can be attributed to the loss of silane and melamine functionalities. Comparing the thermogram of Fe_3_O_4_/Hal-Mel-TEA(IL)-Pd with that of Fe_3_O_4_/Hal-Mel, it was found that the content of IL was about 5 wt%.

In the following, the textural features (specific surface area and average pore size) of Fe_3_O_4_/Hal-Mel-TEA(IL)-Pd were investigated by using BET technique and compared with those of Hal, Fig. S4. It was found that the isotherms of both samples are of type II. The comparison of the specific surface area of the catalyst and Hal showed that the specific surface area of Fe_3_O_4_/Hal-Mel-TEA(IL)-Pd (63 m^2^g^−1^) was higher than that of bare Hal (48 m^2^g^−1^).

**Figure 5:**
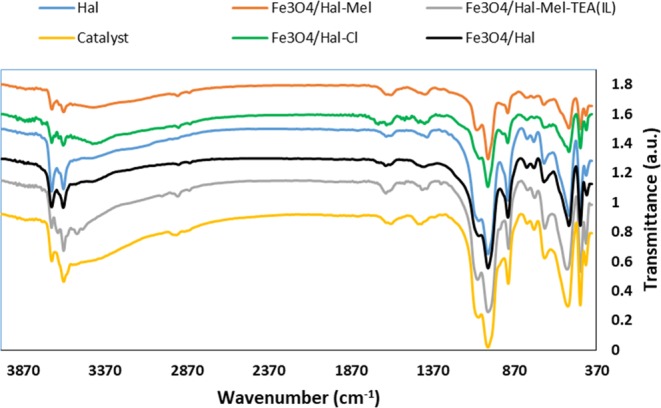
FTIR spectra of Hal, Fe_3_O_4_/Hal, Fe_3_O_4_/Hal-Cl, Fe_3_O_4_/Hal-Mel, Fe_3_O_4_/Hal-Mel-TEA(IL) and Fe_3_O_4_/Hal-Mel-TEA(IL)-Pd.

FTIR spectra of Hal, Fe_3_O_4_/Hal, Fe_3_O_4_/Hal-Cl, Fe_3_O_4_/Hal-Mel, Fe_3_O_4_/Hal-Mel-TEA(IL) and Fe_3_O_4_/Hal-Mel-TEA(IL)-Pd were recorded, Fig. 5. The characteristic bands that represent Hal included the bands at 1091 cm^−1^ (Si-O stretching), 3695 cm^−1^ and 3624 cm^−1^ (inner -OH functionalities) and 534 cm^−1^ (Al-O-Si vibration). The FTIR spectrum of Fe_3_O_4_/Hal showed the characteristic bands of Hal, implying the stability of Hal structure upon magnetization. Notably, the characteristic bands of Fe_3_O_4_ nanoparticles, i.e. the bands at 585 and 635 cm^−1^ (Fe-O stretching) overlapped with the bands of Hal. The FTIR spectrum of Fe_3_O_4_/Hal-Cl is very similar to that of Fe_3_O_4_/Hal. In fact, the characteristic band of silane moiety (Si-O stretching) overlapped with that of Hal. The FTIR spectrum of Fe_3_O_4_/Hal-Mel exhibited the Hal characteristic bands, implying the fact that Hal structure is remained intact in the course of functionalization. Moreover, the band at 1648 cm^−1^ that can be representative of (-C = N) in the melamine structure overlapped with the Hal bands. The FTIR spectra of Fe_3_O_4_/Hal-Mel-TEA(IL) and Fe_3_O_4_/Hal-Mel-TEA(IL)-Pd were similar to that of Fe_3_O_4_/Hal-Mel. Similarly, the characteristic bans of IL overlapped with that of Fe_3_O_4_/Hal-Mel.

Finally, the zeta potential value of Fe_3_O_4_/Hal-Mel-TEA(IL)-Pd was measured and compared with that of Hal. Zeta potential value for pristine Hal was about −39.1 mV, while this value for Fe_3_O_4_/Hal-Mel-TEA(IL)-Pd was −32.4 mV. The reason for the lower zeta potential value of the catalyst can be attributed to the presence of positively charged ammonium salts on the surface of Hal.

### Kinetic and thermodynamic studies

To evaluate the catalytic efficiency of the prepared catalyst, the reduction reaction of MO and RhB dyes was performed in the presence of NaBH_4_ as a reducing agent and the progress of reaction was monitored using UV–visible spectroscopy. To elucidate the necessity of the use of Fe_3_O_4_/Hal-Mel-TEA(IL)-Pd, both dyes were treated with NaBH_4_ for 24 h in the absence of the catalyst. The result confirmed that the reactions did not proceed without the catalyst, indicating the essential role of Fe_3_O_4_/Hal-Mel-TEA(IL)-Pd in the reduction process.

In the following, the effect of the amount of the catalyst on the reduction of MO and RhB was evaluated. To this purpose, reduction of dyes under similar condition in the presence of various amounts of Fe_3_O_4_/Hal-Mel-TEA(IL)-Pd (1, 2, 3, 4 and 5 mg) was carried out. The results, Table S1, showed that in the case of MO, the conversion of the reduction process increased with the increase of the catalyst loading from 1 to 2 mg. However, further increase of the catalyst amount had no effect on the reaction conversion. Regarding RhB, increase of the catalyst amount up to 4 mg led to the increase of the conversion of the reduction process, while further increase of this value to 5 mg did not result in any increase in the reaction conversion, Table S1. Considering these results the optimum catalyst amounts for MO and RhB were found to be 2 and 4 mg respectively.

**Figure 6:**
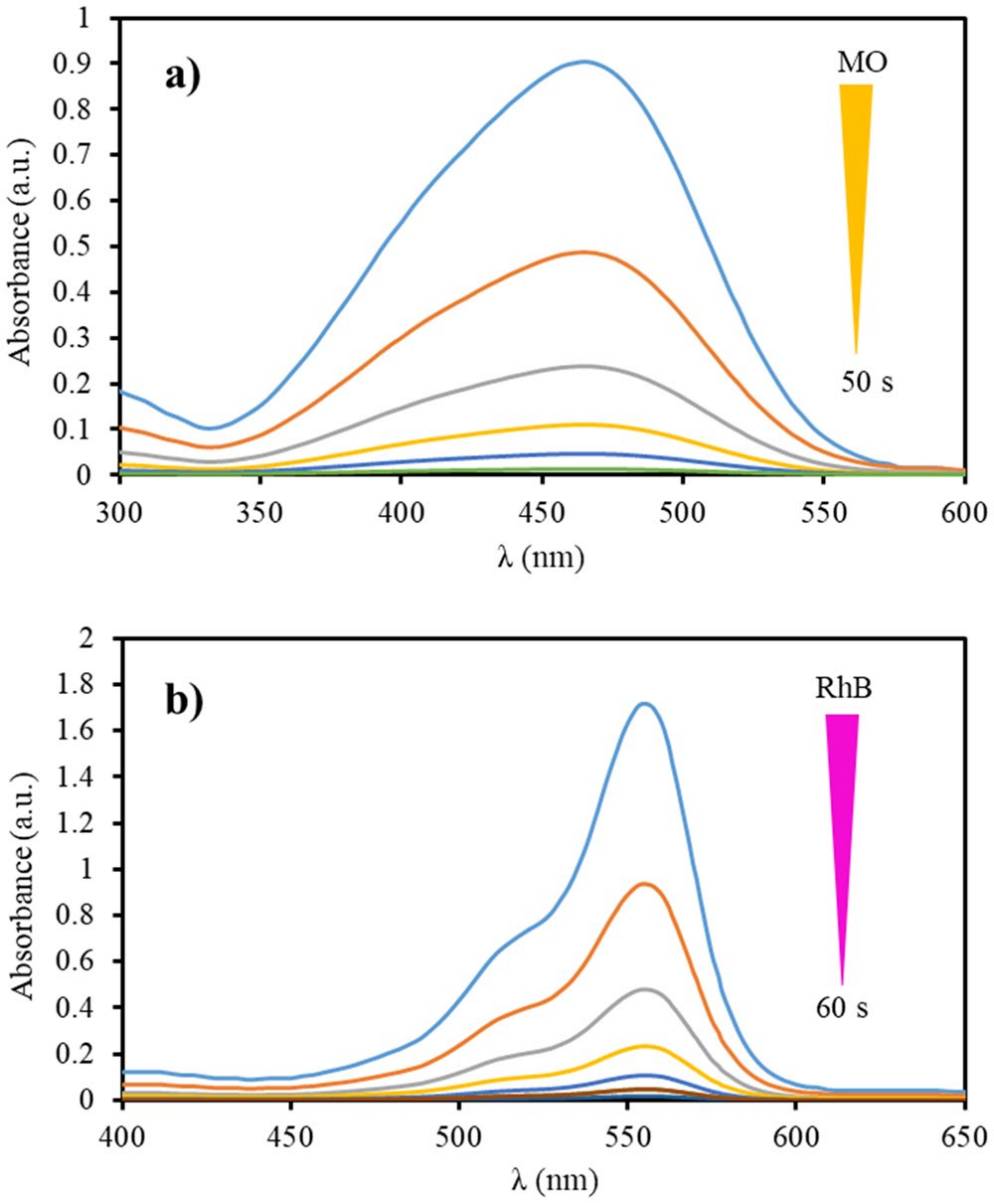
Reduction of MO (**a**) and RhB (**b**) dyes using Fe_3_O_4_/Hal-Mel-TEA(IL)-Pd in aqueous solution recorded by UV-vis spectroscopy.

The temporal UV–vis spectral changes of MO and RhB dyes in the course of the catalytic reduction in the presence of optimum amounts of the catalyst and NaBH_4_ are illustrated in Fig. 6. The decrease of the absorbance at λ_max_ = 465 nm (MO) and λ_max_ = 550 nm (RhB) demonstrated efficient decolorization of dyes in a very short reaction time (50 s for MO and 60 s for RhB). To elucidate whether bare magnetic nanoparticles were catalytically active for the reduction of MO and RhB, the reduction reaction of both dyes in the presence of NaBH_4_ was performed by using 4 mg bare magnetic nanoparticles as catalyst. Monitoring the progress of the reaction certified that within 1 min, MNPs did not promote the reduction of MO and RhB. This result confirmed that in 1 min that is the reaction time, in which the reduction reaction completed in the presence of Fe_3_O_4_/Hal-Mel-TEA(IL)-Pd, MNPs had no role in the reduction reaction, but facilitated the separation of the catalyst from the reaction media.

According to the literature, catalytic reduction of dye by metallic nanoparticles in the presence of excess amount of NaBH_4_ is of pseudo‐first‐order kinetics^31^. The concentration of dye (C_t_) in the course of the reduction can be calculated from the absorbance of the dye at its λ_max_. Accordingly, the rate of the reduction reaction can be measured by considering the decrease of the absorbance of dyes at its λ_max_ versus time. As the ratio of the absorbance of dye at reduction time t = A_t_ to initial time, t = A_0_ is equal to the concentration ratio C_t_/C_0_, the apparent rate constant (k_app_) can be defined by the following equations:

1 $${{\rm{dC}}}_{{\rm{t}}}/{\rm{dt}}=-\,{{\rm{k}}}_{{\rm{app}}}\,{{\rm{C}}}_{{\rm{t}}}$$

2 $${{\rm{lnC}}}_{{\rm{t}}}/{{\rm{C}}}_{0}={{\rm{lnA}}}_{{\rm{t}}}/{{\rm{A}}}_{0}=-\,{{\rm{k}}}_{{\rm{app}}}{\rm{t}}$$

The values of k_app_ for the dyes can be calculated from the slope of ln (A_t_/A_0_) versus t (s) plot. The value of k_app_ obtained at various temperature (298, 303, 308 and 313 K) are reported in Table 1.

**Table 1:** The thermodynamic and kinetic values of the reduction reaction of MO and RhB dyes in the presence of the Fe_3_O_4_/Hal-Mel-TEA(IL)-Pd catalyst.

Dye	T (K)	k (min^−1^)	Ea (kJ/mol)	∆S^#^ (J/mol.K)	∆H^#^ (kJ/mol)
MO	298	0.045	42.02	−139.06	39.40
	303	0.050			
	308	0.067			
	313	0.101			
RhB	298	0.041	39.97	−155.18	34.33
	303	0.067			
	308	0.080			
	313	0.085			

It was found that the kinetic energy of the reactants increased upon increasing of the reaction temperature. This observation can be attributed to the increase of the intensity of the collision of the reactants at elevated temperature. Based on Arrhenius equation (Eq. 3), the activation energies (E_a_) of the reduction reactions of two dyes were estimated by plotting lnk vs. 1/T, Fig. S5 and Table 1. In those plots the R^2^ value for MO and RhB were 0.935 and 0.864 respectively.

3 $$\mathrm{ln}\,{\rm{k}}=\,\mathrm{ln}\,{\rm{A}}-({{\rm{E}}}_{{\rm{a}}}/{\rm{RT}})$$

In this equation E_a_ is activation energy, A stands for Arrhenius factor, T is temperature and R represents ideal gas constant = 8.314 JK^−1^ mol^−1^. In the following, the thermodynamic parameters, i.e. the activation entropy (ΔS^#^) and the activation enthalpy (ΔH^#^) were calculated by using the Eyring equation (Eq. 4).

4 $$\mathrm{ln}({\rm{k}}/{\rm{T}})=\,\mathrm{ln}\,({{\rm{k}}}_{{\rm{B}}}/{\rm{h}})+{\Delta {\rm{S}}}^{\#}/{\rm{R}}-{\Delta {\rm{H}}}^{\#}/{\rm{R}}(1/{\rm{T}})$$

In this equation, k_B_ (Boltzmann constant =1.381 * 10^−23^ JK^−1^) and h (the Planck constant = 6.626 * 10^−34^ JK^−1^ mol^−1^) are constant. Figure S6 shows the plots of ln(k/T) vs. 1/T for MO and RhB. In those plots the R^2^ value for MO and RhB were 0.931 and 0.838 respectively. The entropy values of the reduction reaction of MO and RhB were calculated to be −139.06 and −155.18 Jmol.K^−1^ respectively. Enthalpies of the reduction reactions were measured to be 39.40 and 34.33 kJmol^−1^ for MO and RhB dyes, respectively (Table 1).

To confirm the merit of Fe_3_O_4_/Hal-Mel-TEA(IL)-Pd for the reduction of organic dyes, its performance for the reduction of MO was compared with some of the other catalysts that have been used for the reduction of this dye in the presence of NaBH_4_, Table 2. The low value of k_app_ of Fe_3_O_4_/Hal-Mel-TEA(IL)-Pd compared to the tabulated catalysts showed that Fe_3_O_4_/Hal-Mel-TEA(IL)-Pd can catalyze the reduction of MO rapidly. Moreover, the low value of E_a_ for Fe_3_O_4_/Hal-Mel-TEA(IL)-Pd confirmed the efficiency of this catalyst. The comparison of ∆S^#^ and ∆H^#^ values of the catalyst with the reported ones also indicates that Fe_3_O_4_/Hal-Mel-TEA(IL)-Pd performance can be considered as an efficient catalyst for dye reductive degradation.

**Table 2:** Comparison of the k_app_, Ea, **∆S**^**#**^ and ∆H^#^ values of previously reported catalysts for reduction of MO.

Entry	Catalyst	k_app_ (min^−1^)	E_a_ (kJ/mol)	∆S^#^ (J/mol.K)	∆H^#^ (kJ/mol)	Ref.
1	CSIOAg^a^	6 * 10^−4^ (s^−1^)	76.10	−29.20	73.60	^32^
2	IOAg^b^	NR	102.4	+41	99.9	^32^
3	CuO nanostructure (35 °C)	1 * 10^−2^	59.84	−96.20	57.20	^33^
4	Co:La:TiO_2 (30 ºC)_	7.72 * 10^−3^	NR	−69.15	35.33	^34^
5	TiO_2 (30 ºC)_	5.26 * 10^−3^	NR	−69.76	24.07	^34^
6	**Fe**_**3**_**O**_**4**_**/Hal-Mel-TEA(IL)-Pd**	4.5 * 10^**−2**^ **(s**^**−1**^**)**	**42.02**	**−139.06**	**39.40**	**This work**

a: Ag-coated chitosan-capped γ-Fe_2_O_3_

b: CSIOAg catalyst without chitosan

NR: Not reported.

**Figure 7:**
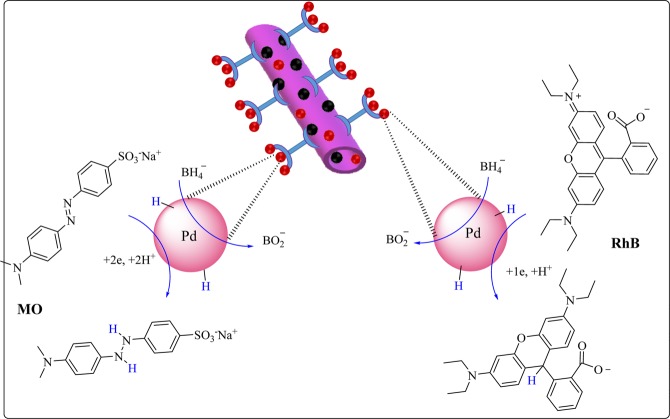
The plausible mechanism for the reduction of dye in the presence of Fe_3_O_4_/Hal-Mel-TEA(IL)-Pd.

The plausible mechanism for the reduction of MO and RhB in the presence of Fe_3_O_4_/Hal-Mel-TEA(IL) is presented in Fig. 7. As shown, sodium borohydride dissociates at the first step of the reaction to produce borohydride ions. Subsequently, BH_4_^−^ ions are adsorbed on the surface of the Pd nanoparticles. Meanwhile, dyes are adsorbed onto the catalyst through π-π stacking interactions. Upon sorption of dyes on Fe_3_O_4_/Hal-Mel-TEA(IL)-Pd, the generated hydride ions transferred to them and dyes will be reduced. In the final step, the reduced dye will be detached from Fe_3_O_4_/Hal-Mel-TEA(IL)-Pd and allows the catalytic cycle to be repeated.

## Reusability

**Figure 8:**
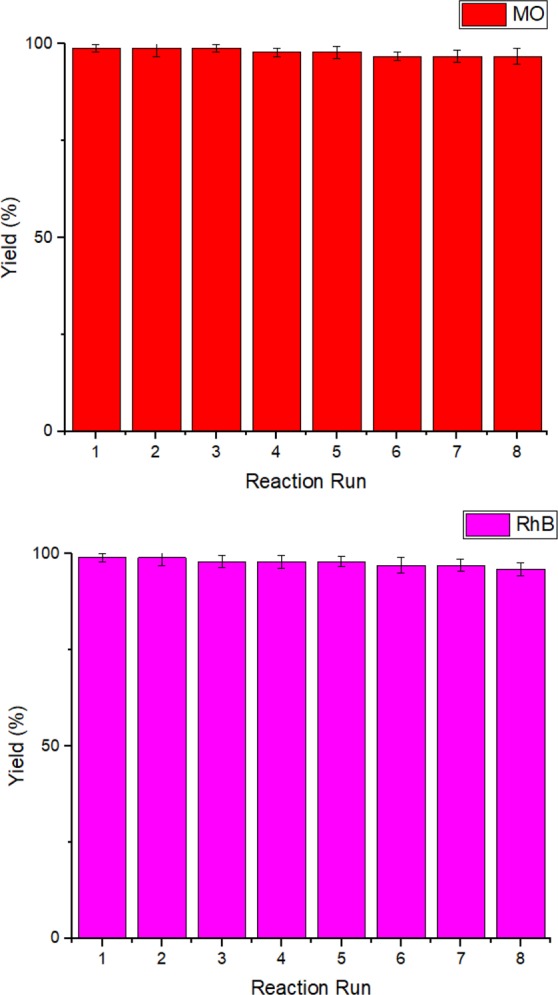
The results of the reusability of Fe_3_O_4_/Hal-Mel-TEA(IL)-Pd for the reduction of MO and RhB.

The reusability of Fe_3_O_4_/Hal-Mel-TEA(IL)-Pd for the reduction of both MO and RhB was investigated. To this purpose, the catalyst was separated from the reaction mixture by using an external magnet and then reused for the next run of the reaction. This cycle was repeated for eight consecutive reaction runs and the obtained yields were measured and compared, Fig. 8. As shown in Fig. 8, Fe_3_O_4_/Hal-Mel-TEA(IL)-Pd exhibited excellent reusability and very slight loss of the catalytic activity after eight reaction runs was observed for both reactions. Encouraged by this result, the Pd leaching of the catalyst was investigated for the catalyst reused for eight reaction runs. Gratifyingly, Pd leaching was insignificant (0.01 wt% of the initial loading).

Next, the stability of Fe_3_O_4_/Hal-Mel-TEA(IL) upon reusing was investigated by recording its FTIR spectrum after eight reuse cycles for the reduction of MO and comparing it with that of fresh Fe_3_O_4_/Hal-Mel-TEA(IL)-Pd, Fig. S7. It was found that the spectrum of the reused Fe_3_O_4_/Hal-Mel-TEA(IL)  was similar to that of the fresh one and no characteristic band of Fe_3_O_4_/Hal-Mel-TEA(IL)-Pd has been disappeared upon reusing.

## Experimental

### Preparation of magnetic Hal

In the first step of the preparation of the catalyst, Hal was magnetized. For this purpose, 2.5 g Hal was dispersed in 120 mL deionized water and the amounts of 1.37 g FeCl_3_. 6H_2_O and 0.5 g FeCl_2_.4H_2_O were added to the Hal suspension. Then, the reaction mixture was stirred at 60 °C. In the following, 10 mL NH_3_ was introduced to the reaction mixture. This mixture was then stirred vigorously for 1 h. At the end of the reaction, the precipitate was collected by an external magnet, washed several times with deionized water and dried at ambient temperature.

### Functionalization of magnetic Hal with CPTES (Fe_3_O_4_/Hal-Cl)

In the next step, magnetic Hal was functionalized with CPTES. More precisely, 1 g magnetic Hal was dispersed in 60 mL dried toluene and then 2.5 mL CPTES was added in a drop wise manner. Subsequently, the mixture was refluxed at 110 °C overnight. Upon completion of the reaction, the product was separated by an external magnet and washed with ethanol and dried at room temperature for 24 h.

### Melamination of Fe_3_O_4_/Hal-Cl (Fe_3_O_4_/Hal-Mel)

First, melamine (5 mmol) was dissolved in DMSO (100 mL). Subsequently, Fe_3_O_4_/Hal-Cl (1 g) was added to melamine solution and the resulting mixture was stirred vigorously and heated up to 100 °C for 24 h. Then, the product, Fe_3_O_4_/Hal-Mel, was collected by external magnet, washed with ethanol several times and dried at ambient temperature overnight.

### Synthesis of Fe_3_O_4_/Hal-Mel-TEA(IL)

At first, Fe_3_O_4_/Hal-Mel (1 g) was dispersed in CH_3_CN by stirring. After that 1, 4 di bromobutane (10 mmol) was added to the aforementioned suspension and the resulting mixture was refluxed for 24 h. Then, the product (Fe_3_O_4_/Hal-Mel-Br) was separated by an external magnet and washed with ethanol and dried at room temperature overnight.

In the next step, 10 mmol of triethanolamine (TEA) was added dropwisely to the suspension of Fe_3_O_4_/Hal-Mel-Br in ethanol. The obtained mixture was then refluxed for 24 h. Finally, the product (Fe_3_O_4_/Hal-Mel-TEA(IL)) was separated by using an external magnet and washed with ethanol and dried at room temperature overnight.

### Synthesis of Fe_3_O_4_/Hal-Mel-TEA(IL)-Pd NPs

To decorate Fe_3_O_4_/Hal-Mel-TEA(IL) with Pd nanoparticles, Fe_3_O_4_/Hal-Mel-TEA(IL) (1 g) was dispersed in deionized water (100 mL). Then, a solution of Pd(OAc)_2_ (1.5 mM) was introduced and the resulting mixture was stirred vigorously at room temperature for 4 h. After that, 10 mL of Heracleum persicum extract was added and the mixture heated at 60 °C overnight. At the end of the reaction, the final product (Fe_3_O_4_/Hal-Mel-TEA(IL)-Pd NPs) was collected by an external magnet, washed with ethanol/water and dried at room temperature overnight. The schematic procedure for the synthesis of the catalyst is illustrated in Fig. 1. According to ICP result, the Pd content of Fe_3_O_4_/Hal-Mel-TEA(IL)-Pd was estimated as 0.93 wt%. Moreover, the content of magnetic nanoparticles was estimated to be 17.6 wt%.

#### Catalytic reduction of dye

To reduce the dyes, dye (2 mL) was dissolved in water and then proper amounts of Fe_3_O_4_/Hal-Mel-TEA(IL)-Pd and NaBH_4_ solution (2 mL, 0.01 M) were added to the reaction mixture. The resulting mixture was subsequently stirred at different temperatures (25, 30, 35 and 40 °C). The progress of the degradation process was monitored by recording the time-dependent UV-vis absorption spectrum of the reaction mixture. At the end of the reaction, Fe_3_O_4_/Hal-Mel-TEA(IL)-Pd was collected magnetically, washed repeatedly with EtOH:H_2_O (1:1) and dried.

## Conclusion

Using Hal and Heracleum persicum extract as natural materials, Fe_3_O_4_/Hal-Mel-TEA(IL)-Pd was prepared through magnetization of Hal followed by surface functionalization and palladation. The composite was then applied as an efficient and magnetically separable catalyst for the reductive degradation of MO and RhB in aqueous media. The catalyst showed excellent catalytic activity for the reduction of both dyes in aqueous media. The activation energy, enthalpy, and entropy for the reduction of methyl orange were estimated as 42.02 kJ mol^−1^, 39.40 kJ mol^−1^, and −139.06 J mol^−1^ K^−1^, respectively. These values  for RhB were calculated as 39.97 kJ mol^−1^, 34.33 kJ mol^−1^, and −155.18 Jmol^−1^K^−1^, respectively. Fe_3_O_4_/Hal-Mel-TEA(IL)-Pd was also highly reusable and could be recovered magnetically and reused for eight reaction runs with only slight loss of its catalytic activity and Pd leaching.

## Supplementary information

Supplementary information.
