# Supplementary material for: Bio-assisted synthesized Pd nanoparticles supported on ionic liquid decorated magnetic halloysite: an efficient catalyst for degradation of dyes
Source: Sci Rep. 2020 Apr 16;10:6535. doi: 10.1038/s41598-020-63558-8 (PMC7162915; doi:10.1038/s41598-020-63558-8)
Supplement: Supplementary file 1 — Supplementary information. [file 41598_2020_63558_MOESM1_ESM.docx]

**Supporting information**

**Bio-assisted synthesized Pd nanoparticles supported on ionic liquid decorated magnetic halloysite: an efficient catalyst for reductive degradation of dyes in aqueous media**

*Samahe Sadjadi*^1^, Pourya Mohammadi^2^, Majid Heravi*^2^*

**Materials:** The chemicals used for the fabrication of the catalyst included Hal, FeCl_3_·6H_2_O, FeCl_2_·4H_2_O, NH_4_OH, (3-chloropropyl) trimethoxysilane (CPTES), melamine, Pd(OAc)_2_, 1,4-dibromobutane and triethanolamine. For the investigation of the catalytic performance of the catalyst, reduction of two dyes, RhB and MO using NaBH_4_ as the reducing agent was targeted. All the reagents were provided from Sigma-Aldrich and used as received. To reduce Pd(II) to Pd(0) Heracleum persicum extract was used.

**Instruments:** The synthesized catalyst, Fe_3_O_4_/Hal-Mel-TEA(IL)-Pd, was characterized using various analyses, including FTIR, TEM, BET, XRD, EDS, VSM, TGA, Zeta potential and ICP-AES. The used apparatus for performing the so-called analyses are as follow: A Siemens, D5000. apparatus was used for recording XRD pattern of Fe_3_O_4_/Hal-Mel-TEA(IL)-Pd. FTIR spectra were obtained using PERKIN-ELMER- Spectrum 65 instrument. The used instrument for studying the morphology of Fe_3_O_4_/Hal-Mel-TEA(IL)-Pd and recording its TEM images was Philips CM30300Kv field emission transmission electron microscope. The magnetic properties of Fe_3_O_4_/Hal-Mel-TEA(IL)-Pd was analyzed using VSM (Lakeshore7407) at ambient temperature. To carry out EDS analysis, Tescan instrument was employed. METTLER TOLEDO thermo gravimetric analysis instrument was used for recording thermogram of the catalyst. The used heating rate was 10 °C min^-1^ in the thermal range of 40 to 800 °C under N_2_ atmosphere. BELSORP Mini II apparatus was applied for recording N_2_-adsorption-desorption isotherm of Fe_3_O_4_/Hal-Mel-TEA(IL)-Pd. Prior to this analysis, Fe_3_O_4_/Hal-Mel-TEA(IL)-Pd was degassed at 423 K for 3 h. The loading of Pd on Fe_3_O_4_/Hal-Mel-TEA(IL)-Pd was measured using ICP analyzer of Vista-pro. Zeta potential measurement was performed in water suspension using a Horiba SZ-100. The progress of reduction of dye was monitored by using UV spectroscopy (PerkinElmer, Lambda 365).

**Figure S1.** Elemental mapping analysis of the catalyst.


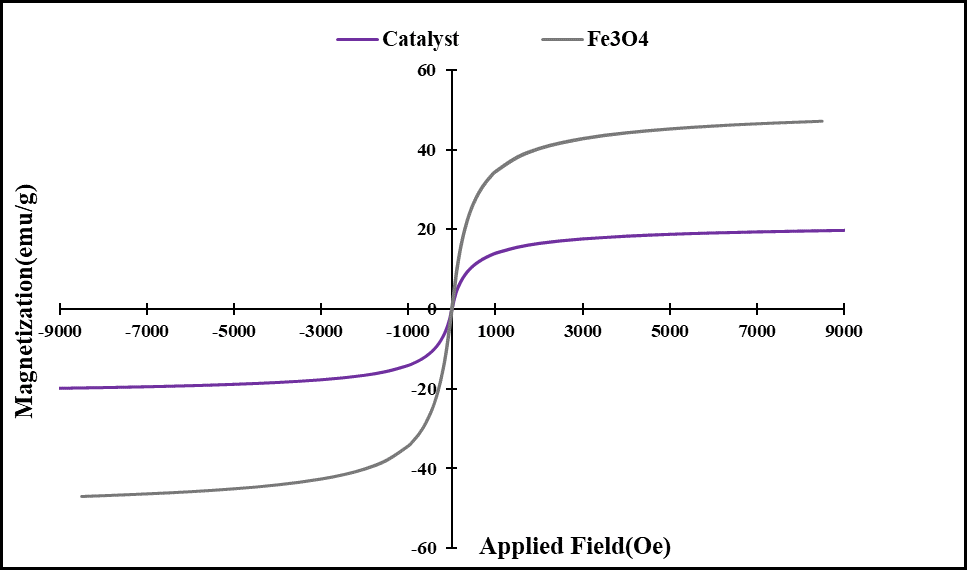


**Figure S2.** VSM analyses of the catalyst and magnetic nanoparticles.


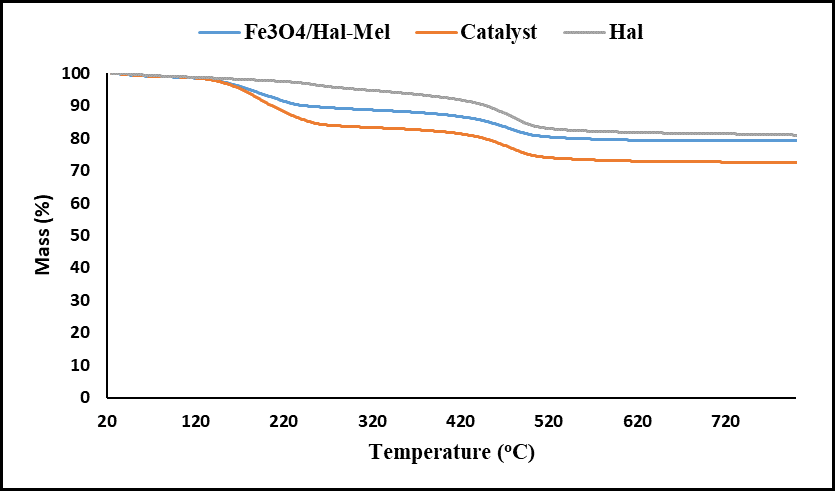


**Figure S3.** Thermograms of Hal, Fe_3_O_4_/Hal-Mel and the catalyst.


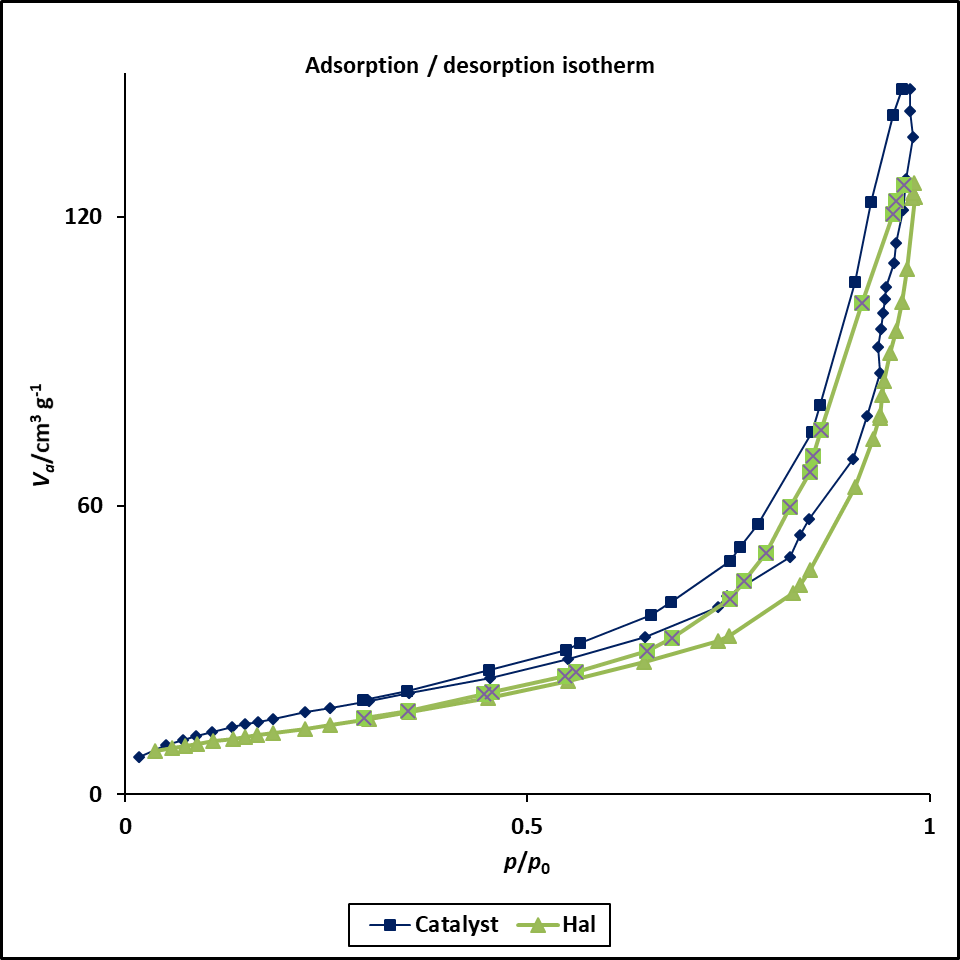


**Figure S4.** N_2_-adsorption desorption isotherm of the catalyst and Hal

**Table S1.** The optimization of the amount of the catalyst for reduction of MO and RhB

| Dye | Catalyst amount (mg)^a^ | Conversion (%) |
| --- | --- | --- |
| MO | 1 | 65 |
|  | 2 | 99 |
|  | 3 | 99 |
| RhB | 1 | 52 |
|  | 2 | 64 |
|  | 3 | 81 |
|  | 4 | 99 |
|  | 5 | 99 |


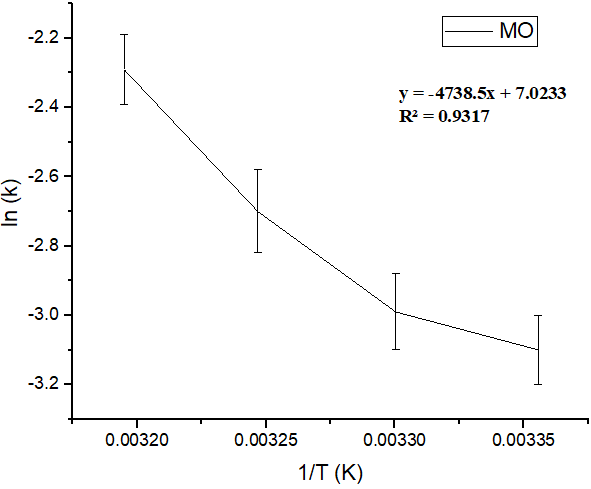


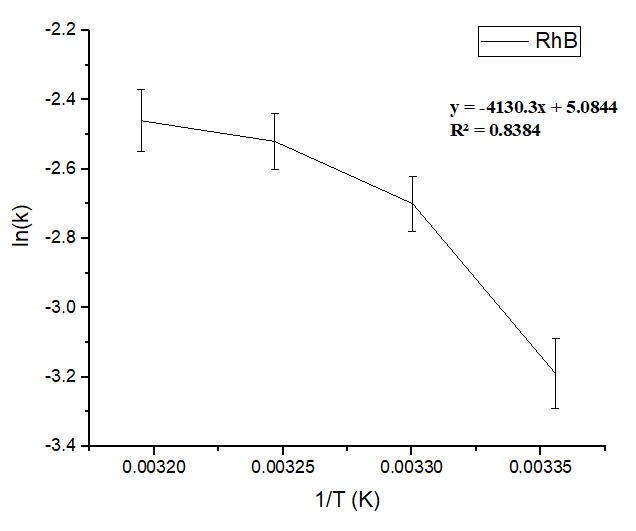


**Figure S5.** The diagram of ln k vs. 1/T (Arrhenius equation) for reduction of MO (a) and RhB (b) dyes at four studied temperatures.


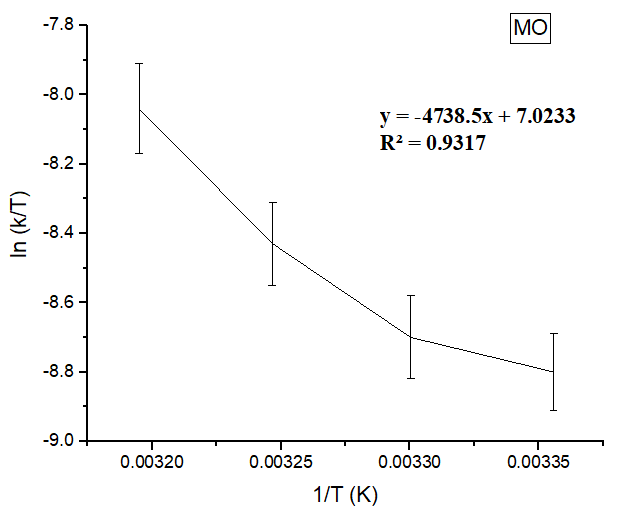


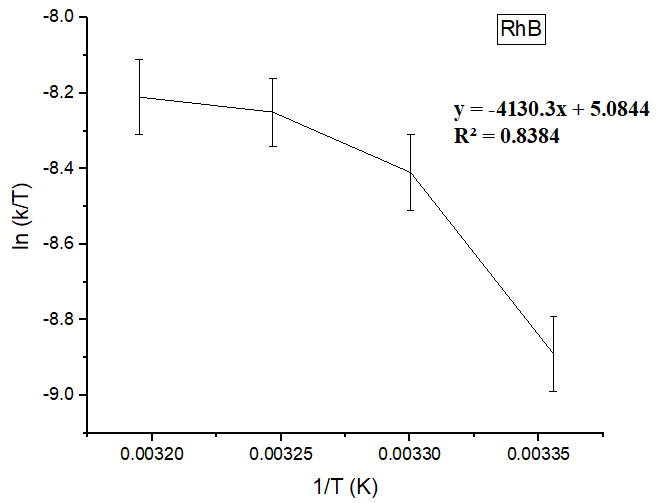


**Figure S6.** The diagram of ln k/T vs. 1/T (Eyring equation) for reduction of MO (a) and RhB (b) dyes at four temperatures.

**
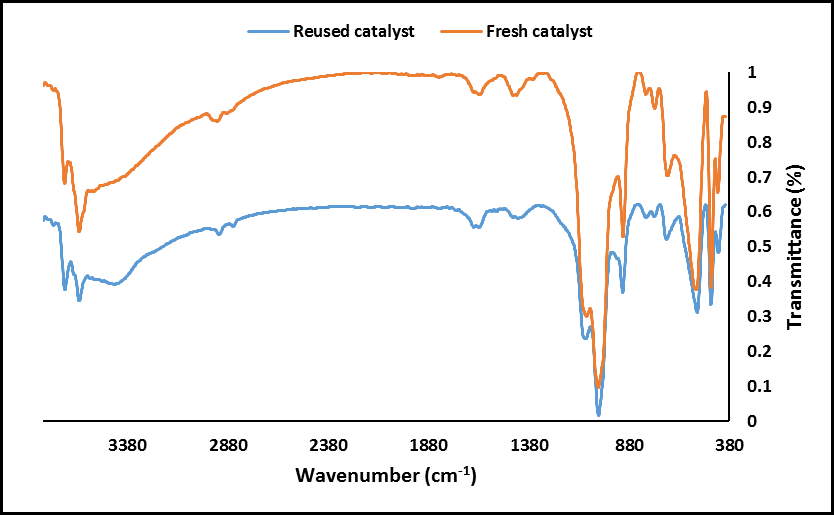
**

**Figure S7.** The FTIR spectra of fresh and recycled Fe_3_O_4_/Hal-Mel-TEA(IL) after eight reaction runs for degradation of MO under optimum reaction condition.
